# Pre- and post-surgical evaluation of the inflammatory response in patients with aortic stenosis treated with different types of prosthesis

**DOI:** 10.1186/s12872-017-0526-1

**Published:** 2017-04-14

**Authors:** Maria Elena Soto, Jose Luis Salas, Jesus Vargas-Barron, Ricardo Marquez, Alejandra Rodriguez-Hernandez, Rafael Bojalil-Parra, Israel Pérez-Torres, Veronica Guarner-Lans

**Affiliations:** 1 Immunology Department, Instituto Nacional de Cardiología “Ignacio Chávez”, Juan Badiano número 1, Colonia Sección XVI, Delegación Tlalpan, Mexico, CP 14080 Mexico; 2 Echocardiography Department, Instituto Nacional de Cardiología “Ignacio Chávez”, Mexico, Mexico; 3 Investigation Department, Instituto Nacional de Cardiología “Ignacio Chávez”, Mexico, Mexico; 4 Intensive care Department, Instituto Nacional de Cardiología “Ignacio Chavez”, Mexico, Mexico; 5 Department of Pathology, Instituto Nacional de Cardiología “Ignacio Chavez”, Mexico, Mexico; 6 Department of Physiology, Instituto Nacional de Cardiología “Ignacio Chávez”, Mexico, Mexico

**Keywords:** Aortic valve, Aortic stenosis, Inflammatory response, Heart surgery

## Abstract

**Background:**

The inflammatory process in aortic valvular stenosis persists after surgery to replace the valve in almost half of the patients. No association has been found to its persistence. The main objective of this study was to evaluate the inflammatory response in patients with aortic stenosis through the determination of several biomarkers in plasma measured before and after the valvular replacement and to seek an association with the type of prosthesis used.

**Methods:**

This is an observational study with a follow up of 6 months in subjects with severe aortic stenosis. Seric concentrations of TNFa, IL-1, IL-6 and ICAM and echocardiographic variables were quantified previous to the surgery and a week and 6 months after it. A group of control subjects paired by age and gender was included.

**Results:**

Seventy-nine subjects were studied of which 57% were male; the average age was of 59 (± 11.4) years. Previous to surgery, the concentration of cytokines was higher in patients than in control subjects. A biological prosthesis was implanted in 48 patients and a mechanical prosthesis in 31. Both, types of prosthesis have components made of titanium. The echocardiograms 1 week and 6 months after the surgery showed a decrease in the mean aortic gradient and an increase in the valvular area (*p* = 0.001). Half of the patients still showed high proinflammatory cytokine levels. There were no differences according to the type of prosthesis implanted after adjustments for demographic variables, comorbidities and echocardiographic data.

**Conclusions:**

The inflammatory response caused by both types of valvular prothesis at 6 months after implantation were similar. Both types of prosthesis are recommended, they had similarities in hemodynamic profiles registered with Doppler echocardiography. Age of the patient or the suitability use of anticoagulants determines the type of prosthesis to be used.

## Background

Aortic stenosis (AS) is a disease that develops with a high local inflammatory response. Its physiopathogeny resembles that of the atherosclerotic process. It is characterized by an initial lesion in the valvular endothelium followed by an active inflammatory infiltrate that leads to progressive calcification, reduction of the valvular area and an increase in the left ventricular (LV) afterload [1–3].

Accordingly, dramatically increased levels of intracellular adhesion molecule 1 (ICAM1) and proinflammatory cytokines such as tumoral necrosis factor-α (TNF-α), interleukin 6 (IL-6) and interleukin 1 (IL-1) are found in patients with AS [4–6]. The only effective treatment in severe AS is the surgical replacement of the aortic valve. However, even after the surgical procedure, the inflammatory state persists in almost half of the patients [7]. However, recently, it has been proposed that the technical approach determines the inflammatory response after surgical and transcatheter aortic valve replacement [8].

Previous studies have not found a correlation between age, gender, smoking habit, ventricular geometry, transvalvular aortic gradient, atherosclerosis risk factors or infection by *Chlamydia pneumoniae* and the persistence of the inflammatory state after the replacement of the aortic valve [9, 10].

The factors triggering or perpetuating the chronic inflammatory process after the valvular replacement are multiple and some of them might even coexist [11]. Therefore, it might be likely that some of the materials with which the different kinds of prosthesis are manufactured could induce the inflammatory mechanisms. [12].

It is well known that mechanical and biological prosthesis are manufactured with different types of materials. In the biological prosthesis, the incomplete process of removing cells from the original animal tissue could lead the presence of protein residues in the valves that might act as immunogens. [13] The metallic components present in mechanical prosthesis such as titanium could also trigger an inflammatory response [14, 15]. It is still unknown if the hemodynamic profile of the patients correlates with the type of inflammatory response or if this response is in correlation with the type of prosthesis implanted.

The main objective of this study was to evaluate the inflammatory response in patients with aortic stenosis through the determination of several biomarkers in plasma measured before and after the valvular replacement and to seek an association with the type of prosthesis used.

## Methods

This is an observational, longitudinal, prospective follow-up study of 6 months. A cohort of patients with aortic stenosis recruited consecutively from September 2013 to January 2015 was studied.

### Inclusion criteria

Subjects of any gender, aged 40 years or more and hospitalized with the diagnosis of severe aortic stenosis determined by nonrheumatic clinical criteria and by echocardiography criteria were included [16]. They agreed to participate by signing an informed consent form.

### Exclusion criteria

Patients with moderate or severe aortic insufficiency, patients having other moderate or severe valvular disease, patients with coronary artery disease, subjects that required other cardiac surgery in addition to aortic valve replacement, patients with autoimmune or oncologic diseases and patients using immunosuppressant were excluded. The use of Non-steroidal anti-inflammatory drugs (NSAIDs) and/or caffeinated foods was suspended 1 day before the blood sampling and 1 week in the case of statins. An elimination criteria was the development of any intra-hospital infection.

### Control group

Forty healthy subjects were matched by age and gender and the age of some controls could be matched with more than one case. Control subjects underwent physical examination, complete transthoracic echocardiography to rule out structural cardiac abnormalities and collection of peripheral blood samples by venopunction for the quantification of inflammatory mediators.

### Sample size

With a confidence of 95% and a power of 90%, a sample of 35 patients per group was estimated by difference in proportions based on the concentration of TNF α obtained in previous studies [10].

### Study design

The study was divided into three phases: preoperative (7 days before surgery), early postoperative (7 days after surgery) and late postoperative (6 months after surgery). In each of the phases an interrogation and physical examination directed to discard active infectious foci was conducted. In the pre-surgical protocol of patients in which cardiac surgery is performed a multidisciplinary team mis involved where the ginecology, odontology and infectology departments intervene. An exhaustive physical examination aimed to determine the clinical diagnosis of active infections is practiced including the performance of an uroculture and nasal and vaginal cultures. In the event of a positive culture, patients are excluded from the surgical programming until they have received antibiotic treatment and a negative culture is obtained. The results from the cultures of every patient previous to surgery were negative and the results from the leucocyte measurement had a mean value of 5600 ± 800. A transthoracic echocardiography study was done and peripheral blood samples for quantification of inflammatory mediators were obtained.

### Determination of cytokines

10 ml of peripheral blood were centrifuged at 2500 rpm/10 min at 4 °C; then 500 ml aliquots of serum were made and stored at −75 °C until cytokine determination. Subsequently, TNF, IL-1, IL-6 and ICAM-1 were determined by enzyme-linked immunosorbent assay (ELISA) sandwich using commercial systems DuoSet (R & D Systems, Minneapolis, MN) according to the instructions provided by the manufacturer [17].

### Echocardiographic study

The echocardiographic study was conducted by two experts’ echocardiographists as is actually recommended [18]. An ultrasound system Philips iE33 xMATRIX (Philips, AndoverMassachusetts) with a transducer ×5 was employed. The LV dimensions were measured using the long parasternal axis; the volumes of the left atrium and LV were calculated with the method of Simpson. The aortic valve area was estimated with the equation of continuity. LV mass was estimated with the method of Devereaux. The mean and maximum aortic transvalvular gradient were obtained from the apical projection of three or five chambers, where an appropriate continuous spectrum from the Doppler was found and where the most transaortic velocity was observed. Systolic pulmonary artery pressure was estimated by the addition of the right atrial pressure to the gradient of tricuspid regurgitation.

### Type of prosthesis implanted

The decision on the type of prosthesis to be implanted was made by the medical team. After aortic valve replacement, the surgery population was divided into two groups according to the type of prosthesis used; biological or mechanical. Biological prostheses manufactured in the National Institute of Cardiology “Ignacio Chavez” from bovine pericardium and titanium metal support were implanted. Mechanical prostheses were bileaflet which are made of pyrolytic carbon and mounted on a support of pyrocarbon covered with Dacron St. Jude Medical™ Masters HP series. A follow up of 6 months from the date of surgery was done.

### Ethical aspects

The study included the signature of an informed consent form that is in accordance with the Declaration of Helsinki and the protocol was approved by the scientific and institutional bioethics committee.

### Statistical analysis

The categorical variables were expressed in proportions, when there was a continuity in mean ± standard deviation or median interquartile range according to distribution. The comparisons were done using Chi Square or Fisher’s exact test for categorical variables; for dimensional variables, Student’s *t* or Mann-Whitney’s U tests were applied. The correlations were done with Pearson’s r for quantitative variables or with Spearman’s rho for qualitative variables. Related samples, where repeated measurements were done, were analyzed with ANOVA and Friedman’s test or with a distribution with a post hoc test to identify differences between groups. The differences were considered as statistically significant when the *p* value was <0.05. The statistical analysis was done with the SPSS program version 22 (SPSS Inc. Chicago, Illinois).

## Results

**Fig. 1:**
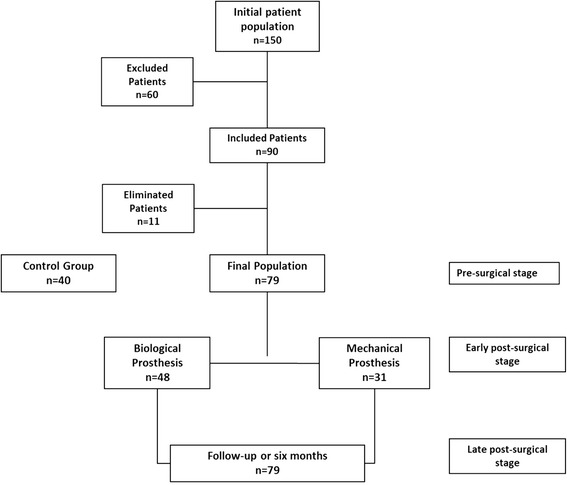
The design of study and flow of patients

We studied 150 consecutive patients with aortic stenosis, of which 90 met inclusion criteria; Patients were eliminated due to the development of serious intrahospital infection (*n* = 2) and to the concomitant finding of arterial coronary disease (*n* = 9), leaving a final population of 79 cases (Fig. 1).

Of the total cases, 45 (57%) were men with an average age of 59 (± 11.4) years, the average age in men with AS was lower (57 ± 12) than in women with AS (62 ± 8.6), *p* = 0.025. Table 1 shows percentage of demograhic characteristics between cases and controls. 10 (12.5%), cases with diabetes, 29(36%) Hypertension and 7 (8.8%) smoking habits. The total prosthesis, biologic and mechanical, implanted were 48(61%) and 31 (39%) by each type of co morbidity were implanted: in patients with ﻿diabetes﻿ 4(8%) and 6(19%), with hypertension 18 (38%) and 11(35%), and with smooking habits 4(8%) and 3 (10%) respectively.

**Table 1:** Demographic characteristics of the AS patient and the control groups

Variables	AS	CONTROL	*p*
Mean (±) SD	*n* = 79	*n* = 40	
*n* (%)			
Age (yr)	59 (11)	58 (11)	0.25
Men	45 (57)	23 (57)	0.68
Women	34 (43)	17 (43)	0.72
Weight (Kg)	69 (11.2)	65 (10)	0.36
Height (cm)	161 (8.8)	165 (9)	0.58
BSA (m^2^)	1.71 (0.17)	1.74 (0.2)	0.87
Diabetes	8 (10)	0	
Hypertension	22 (27.8)	0	

*AS* aortic stenosis, *BSA* body surface area

In the initial echocardiogram all patients had severe aortic stenosis with a valve area of ​​0.67 (± 0.2) cm2, mean aortic gradient of 59 (± 16) mmHg and maximum velocity of flow of 4.8 (± 0.6) m/s. The mean LVEF was 53% (± 17). In 30 patients TNFa concentrations were higher than in controls (38%). In 26 patients (33%) the IL-1 concentrations were elevated and in 30 (38%) there was an elevation of IL-6. All patients had elevated levels of ICAM-1. When comparing the group of patients who had elevated levels of cytokines with those who had normal values no significant differences in age, gender or echocardiographic parameters of aortic stenosis were found. When the comparison between the groups was made for values ​​of inflammatory mediators, there was a tendency to an increase in TNF-α, IL-1, IL-6 and ICAM-1concentrations in AS patients versus controls, with *p* values of 0.08, 0.09, 0.10 and 0.07 respectively (Table 2).

**Table 2:** Echocardiographic variables and interleukin levels of AS patients and control groups

Variables	AS	CONTROL	*p*
mean(±SD)	*n* = 79	*n* = 40	
LV mass (gr/m^2^BSA)	144 (45)	73 (8)	0.001
AoV area (cm^2^)	0.67 (0.2)	2.9 (0.4)	0.001
Ao Mean Gr (mmHg)	59 (16)	2 (0.4)	0.001
Ao Max Vel (m/s)	4.8 (0.6)	0.9 (0.2)	0.001
Ao annulus (mm)	21 (2.6)	22 (3)	NS
LVEF (%)	52 (13)	67 (4.2)	0.001
PASP (mmHg)	36 (13)	22 (2.5)	0.001
TNFα (pg/cc)	0 (0-65)^a^	0 (0-30)^a^	0.08
IL-1 (pg/cc)	0 (0-36)^a^	0 (0-15.7)^a^	0.09
IL-6 (pg/cc)	0 (0-30)^a^	0 (0-10.7)^a^	0.1
ICAM-1 (pg/cc)	3610 (3060-6854)^a^	3555 (3199-3849)^a^	0.07

^a^median (IQR 25-75)

*AS* Aortic stenosis, *LV* left ventricle, *Ao* aortic, *V* valve, *Gr* gradient, *Max* maximum, *Vel* velocity, *Vol* volume, *LVEF* LV expulsion fraction, *PASP* pulmonary artery systolic pressure, *IQR* interquartile range

There were no significant differences in the concentration of cytokines in relation to gender (TNFa: *p* = 0.071, IL-1: *p* = 0.31, IL-6: 0.76 and ICAM-1: *p* = 0.5). As well as no differences in the concentrations of cytokines between the older and younger than 60 years of age subjects (*p* = 0.15, IL-1: *p* = 0.3, IL-6 and ICAM-1 0.2: *p* = 0.9 TNFa).

### Early post surgical stage

In 61% (*n* = 48) of the patients a biological prosthesis was implanted, and in 39% (*n* = 31) a mechanical valve was used. Women are more frequently implanted with a biological prosthesis than men (73% vs 51%), *p* = 0.043. An overall significant improvement in echocardiographic parameters was found with an increase in aortic valve area, a decreased mean aortic transvalvular gradient and a decreased in flow velocity. When the ventricular geometry was analyzed, there was a decrease in LV mass (*p* = 0.001). A significant increase in the concentration of cytokines was found when compared to the preoperative values (Table 3).

**Table 3:** Comparison between surgical stages in all type of prosthesis

Variable mean(±SD)	Preoperative	Early PostCx	LatePostCx	*p*
LV mass (gr/m^2^ BSA)	144 (45)	131 (38)	115 (28)	0.001
AoV area (cm^2^)	0.67 (0.2)	1.5 (0.25)	1.7 (0.15)	0.001
Ao Mean Gr (mmHg)	59 (16)	18 (8)	15 (5.5)	0.001
Ao Max Vel (m/s)	4.8 (0.6)	2.6 (0.6)	2.3 (0.4)	0.01
LVEF (%)	52 (13)	52 (12)	56 (9)	0.01
PASP (mmHg)	36 (13)	34 (9)	28 (5.4)	0.001
TNFα (pg/cc)	0 (0-65)^a^	0 (0-269)^a^	60 (0-204)^a^	0.001
IL-1 (pg/cc)	0 (0-36)^a^	0 (0-90)^a^	0 (0-65)^a^	0.01
IL-6 (pg/cc)	0 (0-30)^a^	18 (0-98)^a^	16 (0-80)^a^	0.048
ICAM-1 (pg/cc)	3610 (3060-6854)^a^	6306 (4984-7058)^a^	3782 (3138-6460)^a^	0.001

^a^median (IQR 25-75)

*BSA* body surface area, *LV* left ventricle, *Ao* aortic, *V* valve, *Vol* volume, *Gr* gradient, *Max* maximum, *Vel* velocity, *LVEF* LV expulsion fraction, *PASP* pulmonary artery systolic pressure, *IQR* interquartile range

### Late post surgical stage

At a follow-up period of 6 months an additional decrease in mean aortic gradient and maximum velocity, and an increase in aortic valve area were observed. There was also a decrease in left ventricular mass (Table 3). About half of the patients still had elevated levels of cytokines at this stage, TNFa: 60% (*n* = 47), IL-1: 42% (*n* = 33) and IL-6: 53% (*n* = 42) and all of the patients maintained high levels of ICAM-1. There was a greater concentration of inflammatory cytokines (TNFa: *p* = 0.001, IL-1: *p* = 0.05, IL-6 and ICAM-1 0003: *p* = 0.039) when compared with the control group. The post-hoc analysis of Friedman test showed that at the late postoperative stage, the cytokine concentrations were higher than before the surgery (TNF: *p* = 0.001, IL-1: *p* = 0.001, IL-6 and ICAM 0.034 1: *p* = 0.001). Comparing the late postoperative stage with the early postoperative stage, there were no significant decreases in the concentrations of TNF (*p* = 0.62) and IL-6 (*p* = 0.24), but a downward tendency in IL-1 (*p* = 0.08) and a significant decrease in ICAM-1 (0016). An inverse correlation was found between ICAM-1 and the maximum aortic velocity (*r* = −0.22, *p* = 0.05) and the mean aortic gradient (*r* = 0.25, *p* = 0.02).

When the population was divided according to the type of prosthesis implanted (biological or mechanical) no significant differences in the parameters of echocardiography were found in the early and late postsurgical stages, nor in the concentration of inflammatory mediators (Table 4).

**Table 4:** Comparison of echocardiographic characteristics and biomarkers among the type of implanted prosthetic aortic valve

	Early Postsurgical			Late Postsurgical		
VARIABLES	Biologic	Mechanic	*p*	Biologic	Mechanic	*p*
mean (±SD)	*n* = 48	*n* = 31		*n* = 48	*n* = 31	
LV mass (gr/m^2^ BSA)	132 (39)	129 (37)	0.75	117 (30)	112 (27)	0.45
AoV area (cm^2^)	1.5 (0.24)	1.5 (0.26)	0.41	1.7 (0.14)	1.7 (0.16)	0.2
Ao Mean Gr (mmHg)	17 (7.9)	19 (8.2)	0.33	15 (5)	17 (6)	0.09
Ao Max Vel (m/s)	2.5 (0.62)	2.7 (0.59)	0.32	2.2 (0.46)	2.4 (0.44)	0.17
LVEF (%)	53 (11)	51 (14)	0.57	56 (8)	56 (11)	0.8
PASP (mmHg)	35 (8)	33 (9)	0.37	29 (5)	28 (6)	0.6
TNFα (pg/cc)	16 (0-264)^a^	0 (0-387)^a^	0.61	0 (60-252)^a^	0 (62-150)^a^	0.41
IL-1 (pg/cm^3^)	9 (0-84)^a^	0 (0-146)^a^	0.7	0 (0-66)^a^	0 (0-65)^a^	0.64
IL-6 (pg/cc)	17 (0-106)^a^	44 (0-92)^a^	0.91	18 (0-78)^a^	0 (0-82)^a^	0.41
ICAM-1 (pg/cc)	6336 (5041-41,060)^a^	6066 (4534-6822)^a^	0.46	4287 (3161-43,985)^a^	3632 (3052-5986)^a^	0.24

^a^median (IQR 25-75)

*BSA* body surface area, *LV* left ventricle, *Ao* aortic, *V* valve, *Gr* gradient, *Max* maximum, *Vel* velocity, *Vol* volume, *LVEF* LV expulsion fraction, *PASP:* pulmonary artery systolic pressure, *IQR:* interquartile range

## Discussion

After surgery for aortic valve replacement, approximately 50% of the patients continued to have an active systemic inflammatory state that was even higher than the one present in the preoperative stage. Our data is consistent with that of previous studies where a significant proportion of subjects remained with high levels of inflammatory mediators [7]. We did not find significant differences in the concentration of cytokines in our population according to the type of valve implanted even when the valve prostheses are manufactured with different materials. Biological valvular prostheses are made from animal tissue that is subjected to a decellularization process and glutaraldehyde fixation in order to reduce their immunogenicity [19, 20]. The artificial valves are manufactured from various synthetic materials, including carbon graphite, pyrolytic carbon and titanium [21]. It has been reported that the residual phospholipids in biological tissue prosthesis could trigger an inflammatory response that might explain the high concentration of cytokines in the subjects who received this type of prosthesis [22]. It is also known that some types of implanted prostheses manufactured with titanium may trigger a chronic inflammatory response, which is not necessarily acute, and where the mechanism might be mediated by Toll receptors [23].

Titanium has high resistance to corrosion in biological environments and it is for this reason that it is the biomaterial of choice for manufacturing implant devices used in various sites in the body. The contact between the biological tissue and the metal promotes the formation of a stable layer of titanium oxide on the surface of the implant [24]. In tissues surrounding the biomaterial and in regional lymph nodes, titanium ions can be found. Despite its biocompatibility there are excellent studies that show that titanium is an allergen capable of triggering an immune response [25].

In experimental mouse models spread of titanium nanoparticles from the intra-articular injection site to the rest of the body has been found [26]. Similarly, neuroinflammation induced by titanium has been found in mice [27]. The results shown here agree with the previously published medical literature. There are studies on inflammation in patients that received mechanical and biological aortic prosthesis where no significant differences between the types of materials were found after an implantation time of 6 months to 2 years similar to those studied here. After some time of the implantation of the biological or mechanical valves, a deposit of extracellular matrix and calcification in the bioprosthetic tissue or the appearance of pannus in the mechanical valves begins, which correlates with prosthetic dysfunction [28].

Oxidative damage and lipid peroxidation was also observed in the synovium of patients with implanted devices which induced the expression of proinflammatory cytokines such as TNFa and IL-1 [29]. Other studies have found toxicity in cells of the heart tissue after exposure to titanium dioxide. There are also well known hiperesensibility reactions to titanium after implantation of this material in dentistry and orthopedics. There are also reports of systemic inflammation caused by titanium nanoparticles [15] and of activation of the immune system with retarded hypersensitivity reactions.

This suggests that the sustained inflammatory response after replacement of the aortic valve may be due to an immune response to components of implanted prostheses such as titanium which is present in both biological and mechanical prosthesis. A striking finding of this study is that the increase in interleukins was similar in patients that had been implanted with any kind of valvular prosthesis. Therefore, we consider that it would be convenient to study the inflammatory response in other types of valvular prosthesis containing titanium and other metallic alloys such as nickel chromium.

The results of this study are consistent with previously published experience. There are studies of inflammation in biological and mechanical aortic prostheses in which no significant differences were found between both manufacturing materials with implantation time from 6 months to 2 years. Problems associated to the durability of the prosthesis and the appearance of dysfunction occurs in a similar rate in mechanical and biological valves. A requirement of another intervention is necessary in up to 60% of the implants for both types of prosthesis [30, 31].

The deposition of extracellular matrix and the formation of pannus leads to the dysfunction of mechanical prostheses. On the other hand, in the dysfunction of biological prostheses calcification and inflammation play an important role [32]. Recent studies have shown that the pathologic calcification in these prosthesis, might be regulated by inductor and inhibitor factors similar to those regulating bone mineralization. The pathophysiology of calcification of biological valves has been evaluated in-vitro and in animal models [33, 34]. For a better comprehension, several aspects should be analyzed such as the determinants of mineralization and their regulating mechanisms. Among these determinants, the metabolism of the host plays an important role. Other mechanical factors include the structure and the chemistry of the implant. Regarding regulation, the pre-treatment of these valves with high doses of glutaraldehide should be considered [35, 36].

Without taking into account the type of prosthesis, inflammation biomarkers have not shown a direct correlation with alterations in the echocardiographic parameters since high levels of cytokines can be found in normal valvular prosthesis and low levels have been found in subjects with dysfunctional ones [37]. This finding was also observed in the present series of patients.

The diversity of the damage mechanisms in each type of prosthesis has been widely discussed and is important when deciding the better therapeutic approach. The decision should take into account the inflammatory, degenerative or calcifying activity of each type of prosthesis. The determination of which type of valve should be used for each patient should include the medical and non-medical conditions, the knowledge of the mechanisms associated to the material to be implanted, and the risk/benefit ratio.

Since the durability of both types of prosthesis is similar, we believe factors such as the cost/effectivity, risk/benefit ratios and quality of life of the patient should be more deeply investigated to decide which type of valve should be implanted.

## Conclusion

The inflammatory response caused by biological valvular prosthesis after 6 months of having been installed is similar to that produced mechanical prostheses. In addition, the similarity in the hemodynamic profiles registered with Doppler echocardiography in patients that had received any of the prosthesis allows us to conclude that both types of valve prostheses are recommended. The decision of the type of valve prosthesis implanted is related more to factors such as age of the patient or the suitability of the use of anticoagulant agents.

## Abbreviations

AS: Aortic stenosis
ICAM: Intercellular adhesion molecules
IL-1: Interleukin 1
IL-6: Interleukin-6
LV: Left ventriculum
NSAIDs: Non-steroidal anti-inflammatory drugs
TNFa: Tumor necrosis factor alfa
